# Manipulation of oil synthesis in *Nannochloropsis* strain NIES-2145 with a phosphorus starvation–inducible promoter from *Chlamydomonas reinhardtii*

**DOI:** 10.3389/fmicb.2015.00912

**Published:** 2015-09-07

**Authors:** Masako Iwai, Koichi Hori, Yuko Sasaki-Sekimoto, Mie Shimojima, Hiroyuki Ohta

**Affiliations:** ^1^Graduate School of Bioscience and Biotechnology, Tokyo Institute of TechnologyYokohama, Japan; ^2^JST CRESTTokyo, Japan; ^3^Earth-Life Science Institute, Tokyo Institute of TechnologyTokyo, Japan

**Keywords:** algae, *Nannochloropsis*, phosphorus starvation, inducible promoter, triacylglycerol

## Abstract

Microalgae accumulate triacylglycerols (TAGs) under conditions of nutrient stress. Phosphorus (P) starvation induces the accumulation of TAGs, and the cells under P starvation maintain growth through photosynthesis. We recently reported that P starvation–dependent overexpression of type-2 diacylglycerol acyl-CoA acyltransferase (CrDGTT4) from *Chlamydomonas reinhardtii* using a sulfoquinovosyldiacylglycerol synthase 2 (SQD2) promoter, which has increased activity during P starvation, enhances TAG accumulation in *C. reinhardtii* cells. As a result, the content of C18:1 fatty acid, a preferred substrate of CrDGTT4, is increased in TAGs. Here we isolated genes encoding SQD2 from strain NIES-2145 of the eustigmatophyte *Nannochloropsis* and showed that their expression, like that in *C. reinhardtii*, was up-regulated during P starvation. To enhance oil accumulation under P starvation, we transformed pCrSQD2-CrDGTT4 into *Nannochloropsis* strain NIES-2145. The transformants had a fatty acid composition that was more similar to that of *C. reinhardtii*, which resulted in enhanced TAG accumulation and higher 18:1(9) content. The results indicated that the P starvation–inducible promoter of *C. reinhardtii* was able to drive expression of the *CrDGTT4* gene in *Nannochloropsis* strain NIES-2145 under P starvation. We conclude that the heterologous *CrSQD2* promoter is effective in manipulating TAG synthesis in *Nannochloropsis* during P starvation.

## Introduction

Algal biofuel technology exploits algal photosynthesis and biosynthesis processes to produce oils using only sunlight, CO_2_, water and limited nutrients. Many microalgae accumulate triacylglycerols (TAGs) during nutrient stress (Giroud et al., 1988; Guschina and Harwood, 2006; Hu et al., 2008). It is estimated that the annual oil production from algae is in the range of 40,700–53,200 L ha^−1^ year^−1^ (Weyer et al., 2010). Therefore, the potential use of microalgae to provide biofuel feedstock is receiving significant attention.

A eukaryotic microalga, *Chlamydomonas reinhardtii*, is a model organism for studying algal biodiesel production because of its available whole-genome sequence and the ability to manipulate gene expression within this organism (Harris, 2009). Under stress conditions, such as nitrogen (N) starvation, *Chlamydomonas* nearly stops its growth and accumulates large amounts of TAGs (Grossman, 2000; Zhang et al., 2004). We reported that *C. reinhardtii* cells in logarithmic growth phase that were diluted into fresh medium showed substantial TAG accumulation under N or P deprivation (Iwai et al., 2014). P deprivation substantially induced the accumulation of oil droplets in the cytosol but allowed the maintenance of thylakoid membranes in *C. reinhardtii* (Iwai et al., 2014).

*Nannochloropsis* species are unicellular photosynthetic microalgae in the class Eustigmatophyceae. Previously known as “marine Chlorella,” this class was identified on the basis of its ultrastructure and named *Nannochloropsis* by Maruyama et al. (1986). Its cells are spherical to slightly ovoid, are 2–4 μm in diameter and contain ovoid or cup-shaped chloroplasts (Maruyama et al., 1986). *Nannochloropsis* are of interest because of their rapid growth and their ability to produce large quantities of TAGs and be cultured on an industrial scale (Hodgson et al., 1991; Rodolfi et al., 2009; Huerlimann et al., 2010) In recent years, *Nannochloropsis* strains have been studied for their biomass production and their lipid composition and content under different growth conditions (Hu and Gao, 2006; Converti et al., 2009; Rodolfi et al., 2009; Simionato et al., 2011; Arudchelvam and Nirmalakhandan, 2012; Vieler et al., 2012b; Wang et al., 2014). In *Nannochloropsis* strains, N deprivation induces lipid accumulation and lipid droplet formation (Rodolfi et al., 2009; Vieler et al., 2012a,b; Martin et al., 2014), and their lipid content also increases with decreasing P concentrations (Hu and Gao, 2006; Rodolfi et al., 2009; Bondioli et al., 2012).

Diacylglycerol acyltransferase (DGAT) catalyzes the last step of TAG synthesis involving *sn*-1,2 diacylglycerol and acyl-CoA (Lung and Weselake, 2006; Rajakumari et al., 2008) and includes two major types, type 1 DGAT (DGAT1) and type 2 DGAT (DGAT2) (Cases et al., 1998; Lardizabal et al., 2001; Shockey et al., 2006). There are six *DGAT* genes in *C. reinhardtii* (encoding one *DGAT1* and five *DGAT2*s) (Miller et al., 2010; Boyle et al., 2012). In contrast, 12 or 13 *DGAT* genes (encoding one or two *DGAT1*s and 11 *DGAT2*s) are present in *Nannochloropsis* strains (Radakovits et al., 2012; Vieler et al., 2012b; Wang et al., 2014). During N starvation in *Nannochloropsis oceanica* IMET1, seven DGAT transcripts are up-regulated and six other DGAT transcripts are down-regulated (Li et al., 2014).

Endogenous promoters are mainly used for the nuclear transformation of algae such as *C. reinhardtii* (Eichler-Stahlberg et al., 2009; Harris, 2009; Brueggeman et al., 2014), although stable nuclear transformation of algae is feasible with promoters from heterologous sources that are categorized in the same class (Hirata et al., 2011; Lerche and Hallmann, 2013, 2014). Transformation experiments using *Nannochloropsis* with endogenous promoters have recently been reported (Kilian et al., 2011; Vieler et al., 2012b). Additionally, a construct with the *C. reinhardtii* α-tubulin promoter was used for transforming *Nannochloropsis* (Vieler et al., 2012b). However, the stable nuclear transformation of *Nannochloropsis* using an inducible promoter from a heterologous source under specific conditions, in particular an oil-accumulating condition such as nutrient starvation, has not been reported.

In this study, we found that *Nannochloropsis* strain NIES-2145 has a homolog of the *Arabidopsis SQD2* gene. Our results suggest that there is a common expression control system in a wide range of algal species, including primary and secondary microalgae, for adaptation to low P. We used the promoter of *sulfoquinovosyldiacylglycerol (SQDG) synthase 2 (SQD2)*, which encodes the sulfoquinovosyl transferase that catalyzes the second step of sulfolipid biosynthesis (Yu et al., 2002). The transcript levels of the *SQD2* gene homologs are increased in *C. reinhardtii* and *Arabidopsis* by P starvation concomitant with increases in the SQDG content (Yu et al., 2002; Chang et al., 2005; Okazaki et al., 2009; Iwai et al., 2014). The promoter of *SQD2* induced expression of the down stream gene during P starvation, to successfully overexpress *C. reinhardtii* type-2 diacylglycerol acyl-CoA acyltransferase (CrDGTT4) in *Nannochloropsis* strain NIES-2145 under P starvation. The total lipid content, neutral lipid content and fatty acid profiles were determined. CrDGTT4 enhanced TAG accumulation under P starvation by changing the fatty acid composition to be more similar to that of *C. reinhardtii*. Therefore, the heterologous Cr*SQD2* promoter is effective in manipulating oil synthesis in *Nannochloropsis* during P starvation.

## Materials and methods

### Materials and culture conditions

*Nannochloropsis* strain NIES-2145 was obtained from the Microbial Culture Collection of the National Institute for Environmental Studies, Japan. *Nannochloropsis* strain NIES-2145 was grown photoautotrophically in f/2 medium (Guillard and Ryther, 1962) or F2N50%SW medium (standard medium), which is F2N medium (Kilian et al., 2011) made with 50% artificial seawater (Wako Pure Chemical Industries, Ltd., Japan). Liquid cultures were grown in continuous white light (20–40 μmol photons m^−2^ s^−1^) at room temperature. Nutrient deficiency was induced by centrifuging the cells for 10 min at 2000 × g, washing them twice with the respective medium and subsequently resuspending them in the standard medium without NaNO_3_ and NH_4_Cl (–N) or phosphate (–P) solutions. Agar plates were prepared using 0.8% Bacto agar (Difco, USA) in standard medium. The cells were maintained on these plates at the same light intensity at room temperature.

### Analysis of differential gene expression levels

The differential expression levels of the *CrDGAT* gene and *SQD2* genes in *Nannochloropsis* strain NIES-2145 cultured in standard, -P and -N media were determined using quantitative real-time PCR (qPCR). RNA was extracted from standard, -P and -N cultures using the phenol/chloroform method. Total RNA (500 ng) was used for the synthesis of cDNA with an oligo(dT)_18_ primer, random hexamers and Superscript II reverse transcriptase (Invitrogen, Carlsbad, CA). cDNA was amplified using SYBR Premix Ex Taq II (Takara, Japan). The Thermal Cycler Dice Real Time System and Multiplate RQ software (Takara) were used for the analysis. Expression levels of the *CrDGAT* gene and *SQD2* genes were normalized to TUB mRNA expression. The *CrDGTT4* expression levels in *Nannochloropsis* cultured in -P medium were compared with those of *Nannochloropsis* cultured in the standard medium (the values of which were set to 1). Primers are listed in Supplemental Table 1.

### Construction and identification of *CrDGTT4*-overexpressing NIES-2145 cell lines

Transformation experiments were performed using a construct developed for the nuclear transformation of *Nannochloropsis*, pMD20 with a NT7 cassette (Kilian et al., 2011), except that a truncated bidirectional violaxanthin–chlorophyll a binding protein 2 promoter and the violaxanthin–chlorophyll a binding protein 1 3′ UTR from *Nannochloropsis* strain NIES-2145 were used and that the *Eco*RV restriction site was inserted between the ble gene and the violaxanthin–chlorophyll a binding protein 1 3′ UTR. To construct the expression plasmid carrying the *CrDGTT4* (XM_001693137.1) gene, pCrSQD2aDGTT4, which contains the *C. reinhardtii SQD2* promoter region and the coding sequence of the *DGTT4* gene (Iwai et al., 2014), was digested with restriction endonucleases *Eco*RV and *Spe*I and was treated using a DNA Blunting Kit (Takara). The resulting blunt-ended DNA was cloned into pMD-NT7, which was digested with restriction endonuclease *Eco*RV. Electroporation with a supplier was then used to transform the nuclear genome of *Nannochloropsis* strain NIES-2145 cells with 2–10 μg vector construct (Vieler et al., 2012b). The selection of transformants was performed on standard medium supplemented with 2 μg mL^−1^ Zeocin. Approximately 100 positive transformants were identified by growth on the selective medium, and overexpression levels in each resulting line were determined using qPCR.

### Extraction and separation of lipids

*Nannochloropsis* cells were harvested by centrifugation (10 min at 3000 × g), and total lipids were extracted as described (Bligh and Dyer, 1959). The lipids were dissolved in chloroform/methanol (2:1, v/v) and stored at −20°C. Lipid classes were separated using two-dimensional thin-layer chromatography (TLC). The first dimension was developed using chloroform/methanol/7 N ammonia water (115:80:8, v/v/v). After the plates were dried for 1 h, the second dimension was developed using chloroform/methanol/acetic acid/water (170:25:15:3, v/v/v/v). TAGs were separated by TLC using the solvent system hexane/diethyl ether/acetic acid (160:40:4, v/v/v). Lipids were visualized under UV light after spraying the TLC plates with 0.001% (w/v) primuline in 80% (v/v) acetone.

### Preparation of fatty acid methyl esters (FAMEs)

Each lipid was scraped off of the TLC plates. FAMEs were obtained by incubating lipids for 1 h at 85°C in the presence of 5% (v/v) hydrogen chloride–methanol solution (Wako Pure Chemical Industries) (Iwai et al., 2014). FAMEs were extracted using hexane and were determined by gas chromatography (GC). Fatty acid quantification was performed by comparing samples with the FAMEs derived from the internal standard, pentadecanoic acid (15:0).

### GC analysis

FAMEs were analyzed using a GC-2014 (Shimadzu Corporation, Kyoto, Japan) with a HR-SS-10 capillary column (length, 25 m; internal diameter, 0.25 mm; Shinwa Chemical Industries, Ltd., Japan). The fatty acid profiles were determined by comparison with a standard reference mix composed of FAMEs (Supelco 18917-1AMP, 18913-1AMP, CRM47885; Sigma-Aldrich, GLC411, GLC462; Funakoshi, Japan).

### GC-mass spectrometry (MS)

The qualitative composition of FAMEs was studied using a GS-MS (model GCMS-TQ8030; Shimadzu Corporation). High-grade pure helium was used as the carrier gas. The ionization voltage was 70 eV, and the ionization temperature was 200°C. Mass spectra were scanned every 0.2 s. For the analysis of the FAMEs, a DB-5 ms column (length, 30 m; internal diameter, 0.25 mm; Agilent Technologies, Inc., CA, USA) was used. For each sample, 1 μL was injected on the column into a helium gas flow held constant at 1.4 mL min^−1^. The column temperature was elevated from 40°C to 320°C at a rate of 6°C min^−1^ and then kept at 320°C for 1.15 min.

### *SQD2* cDNA

RNA was extracted from standard and -P cultures using the phenol/chloroform method. Total RNA (500 ng) was used for the synthesis of cDNA with an oligo(dT)_18_ primer, random hexamers and Superscript II reverse transcriptase (Invitrogen, Carlsbad, CA). The primers used are listed in Supplemental Table 1. The gene sequences were then cloned into the pMD20 vector (Takara) for DNA sequence.

### Phylogenetic analyses

Amino acid sequences were aligned using MAFFT v7.220 (Katoh and Standley, 2013). Gblocks 0.91b (Talavera and Castresana, 2007) was used to remove any poorly conserved regions. The phylogenetic analyses were performed using the maximum likelihood and the neighbor-joining methods in MEGA6 (Tamura et al., 2013), and a Bayesian analysis in MrBayes 3.2.3 (Ronquist et al., 2012). The amino acid substitution model for the maximum likelihood and Bayesian inference methods was selected using Aminosan (Tanabe, 2011). The maximum likelihood and neighbor-joining methods were performed based on the LG model + Gamma (eight categories) with 1000 bootstraps and the JTT model with 1000 bootstraps, respectively. The Bayesian analysis was performed based on the LG model + Gamma (eight categories) for 1,000,000 generations. Every 500th generation was sampled, and the first 200 trees were discarded as burn-in.

### Motifs analysis

The analysis of the motifs was performed *in silico* using New PLACE (https://sogo.dna.affrc.go.jp/cgi-bin/sogo.cgi?sid=&lang=en&pj=640&action=page&page=newplace), a database of *cis*-acting regulatory DNA elements from plants, to find the binding motifs of transcription factors.

## Results

### Cell growth and tag accumulation in cells under P deprivation

**Figure 1:**
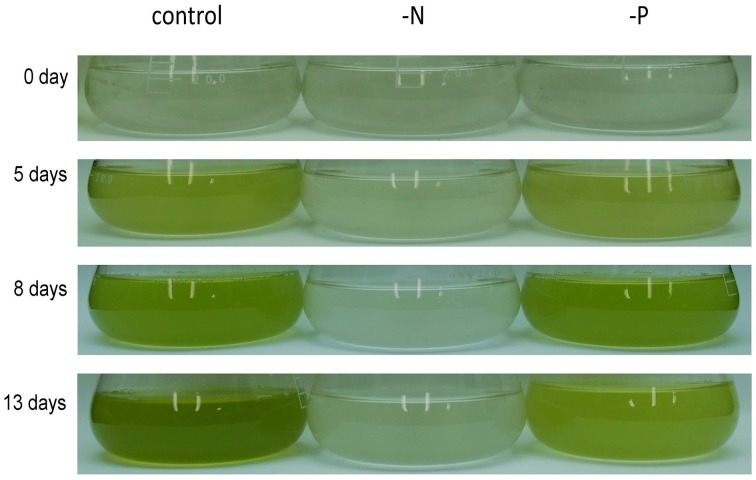
**Growth of ***Nannochloropsis*** NIES-2145 under standard growth conditions, N starvation and P starvation**. *Nannochloropsis* cells cultured to the logarithmic phase were inoculated into standard (control), -N and -P media. Cells were cultured for the indicated times after transfer before being photographed.

**Figure 2:**
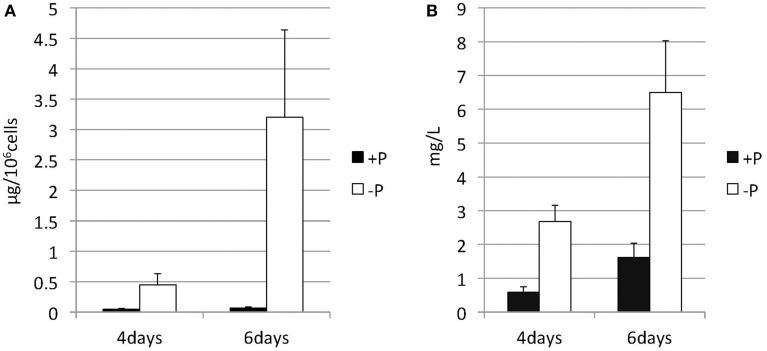
**Changes in the TAG content of ***Nannochloropsis*** in response to P starvation**. Cells cultured to a logarithmic phase under standard conditions were then inoculated into standard (+P) or -P medium and cultured for 4 or 6 days. The y-axis is **(A)** Total TAG per 10^6^ cells or **(B)** total TAG per liter of culture. Values represent the mean from three independent experiments ± SD.

*Nannochloropsis* strain NIES-2145 was cultured to the logarithmic phase in f/2 medium. Cultures were then centrifuged and resuspended in fresh f/2 or in N-depleted (-N) or P-depleted (-P) f/2 medium. The cultures derived from logarithmic-phase cultures were inoculated at low cell densities (1 × 10^6^ cells mL^−1^). Under N-deprived conditions, the *Nannochloropsis* cells were nearly white, but the cells exposed to P starvation were still green (Figure 1). Because *C. reinhardtii* cells diluted into P-depleted medium show substantial TAG accumulation (Iwai et al., 2014), we investigated whether TAG was accumulated in these nutrient-starved cells by quantifying lipid-derived FAMEs using GC. Cells had substantially increased TAG levels under both N and P deprivation (Supplemental Figure 1). Compared with cells grown in complete medium, the N-starved cells contained ~11-fold and the P-starved cells contained ~6-fold more TAG per cell after 13 days. The TAG accumulation in the N-starved cells is consistent with that in recent reports (Bondioli et al., 2012; Vieler et al., 2012b; Simionato et al., 2013). When the cultures were inoculated at 5 × 10^6^ cells mL^−1^ in standard medium or –P medium, P-starved cells contained more TAG per cell than the corresponding cells maintained in the standard medium (~7-fold more at 4 days and ~47-fold more at 6 days; Figure 2A). P-starved cultures contained ~4.6-fold more TAG per liter at 4 days and ~4-fold more TAG per liter at 6 days than the corresponding culture maintained in the standard medium (Figure 2B).

### Increase in SQDG and SQD2 homolog promoter activity during P starvation

**Figure 3:**
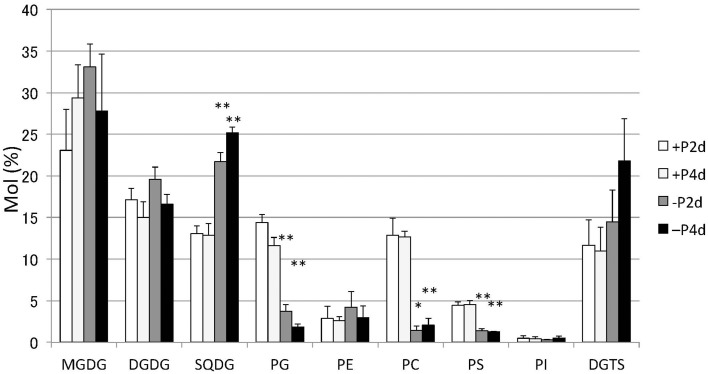
**Changes in major lipid classes in P-starved cells**. Cells cultured to a logarithmic phase under standard conditions were then inoculated into standard (+P) or -P medium and cultured for 2 days (2 d) or 4 days (4 d). Lipid levels were determined by GC. Values represent the mean from three independent experiments ± SD. Asterisks indicate a statistically significant difference compared with the empty vector control based on a two-tailed Student's *t*-test (^*^P < 0.05 and ^**^P < 0.01).

Lipids extracted from *Nannochloropsis* strain NIES-2145 were separated by two-dimensional TLC, followed by GC-flame ionization detection and GC-MS. P starvation caused significant changes in lipid composition. The glycerolipid composition of *Nannochloropsis* strain NIES-2145 resembles that of the photosynthetic organism *Arabidopsis thaliana* (Li-Beisson et al., 2010), being comprised mostly of the prevalent glycoglycerolipids monogalactosyldiacylglycerol (MGDG), DGDG and SQDG, as well as the common phospholipids phosphatidylcholine (PC), phosphatidylethanolamine (PE), phosphatidylserine (PS), phosphatidylinositol (PI), and phosphatidylglycerol (PG). In addition, a betaine lipid diacylglycerol-*N*,*N*,*N*-trimethylhomoserine (DGTS) is present, as in *C. reinhardtii* (Sato and Furuya, 1985; Giroud et al., 1988). P deprivation resulted in elevated levels of SQDG and DGTS. PG, PC, and PS which contain phosphates in their head groups, decreased after P deprivation (Figure 3). This result corroborates previous reports on P deprivation in several species (Benning et al., 1993; Essigmann et al., 1998; Sato et al., 2000; Khozin-Goldberg and Cohen, 2006).

**Figure 4:**
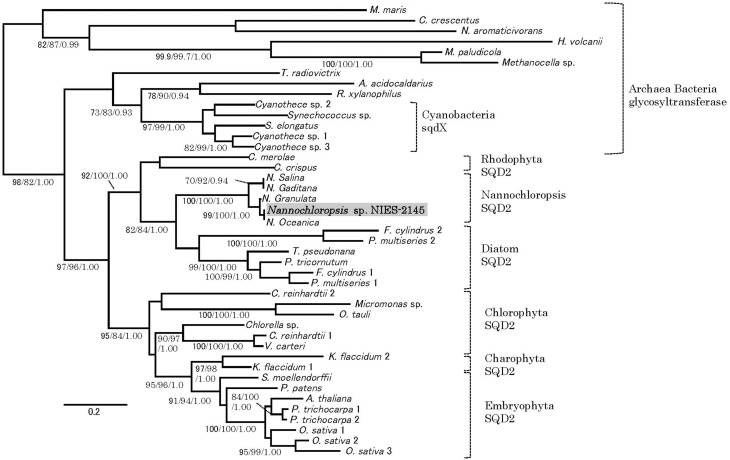
**Phylogenetic tree analysis of SQD2**. A phylogeny incorporating eukaryotic SQD2, cyanobacterial sqdX and the glycosyl transferase proteins of other organisms was constructed using Bayesian inference, maximum likelihood and neighbor-joining algorithms. It is based on a protein comparison. The topologies and branch lengths were calculated using maximum likelihood. Bootstrap values (maximum likelihood and neighbor-joining) higher than 70 and Bayesian posterior probabilities (Bayesian inference) higher than 0.9 are indicated under each branch (maximum likelihood/neighbor-joining/Bayesian inference). The scale bar represents 0.2 amino acid substitutions per site. Sequence accession numbers or sequence resources for the phylogenetic tree are as follows: Archaea glycosyl transferase: *Methanocella paludicola* (YP_003355097), *Methanocella* sp. (YP_685710) and *Haloferax volcanii* (YP_003535220); Alpha-proteobacteria glycosyl transferase: *Caulobacter crescentus* (YP_002516166), *Novosphingobium aromaticivorans* (WP_011446186), and *Maricaulis maris* (WP_011642337); Cyanobacteria sqdX: *Cyanothece* sp. 1 (WP_015784192), *Cyanothece* sp. 2 (WP_012629991), *Synechococcus elongatus* (WP_011243256), *Synechococcus* sp. (WP_011429929), and *Cyanothece* sp. 3 (WP_015956855); Other bacterial glycosyl transferases: *Truepera radiovictrix* (WP_013178429), *Rubrobacter xylanophilus* (WP_011564279), and *Alicyclobacillus acidocaldarius* (WP_012810695); Rhodophyta SQD2: *Chondrus crispus* (XP_005715110), and *Cyanidioschyzon merolae* (XP_005538341); Stramenopile SQD2: *Nannochloropsis* sp. NIES-2145 (DDBJ Accession LC061442), *Nannochloropsis oceanica* IMET1 (BioProject: PRJNA202418, scaffold00247.g6721), *Nannochloropsis granulata* CCMP529 (BioProject: PRJNA65111, evm.model.NODE_3998_length_11613_cov_18.861534.4), *Nannochloropsis salina* CCMP537 (BioProject: PRJNA62503, evm.model.NODE_9973_length_179428_cov_24.782236.3), *Nannochloropsis gaditana* (EWM27152), *Phaeodactylum tricornutum* (JGI Protein ID: 50356), *Fragilariopsis cylindrus* 1 (JGI Protein ID: 207999), *Fragilariopsis cylindrus* 2 (JGI Protein ID: 158007), *Pseudo-nitzschia multiseries* 1 (JGI Protein ID: 252457), *Pseudo-nitzschia multiseries* 2 (JGI Protein ID: 145536), *Thalassiosira pseudonana* (JGI Protein ID: 38775), [Chlorophyta] *Ostreococcus tauri* (JGI Protein ID: 3203), *Micromonas* sp. (JGI Protein ID: 58169), *Chlorella* sp. (JGI Protein ID: 33086), *Volvox carteri* (Phytozome Transcript Name: Vocar20009459m), *Chlamydomonas reinhardtii* 1 (Phytozome Transcript Name: Cre01.g038550.t1.3), and *Chlamydomonas reinhardtii* 2 (Phytozome Transcript Name: Cre16.g689150.t1.2); Charophyta: *Klebsormidium flaccidum* 1 (BioProject: PRJDB718, kfl00392_0070) and *Klebsormidium flaccidum* 2 (BioProject: PRJDB718, kfl00041_0140), and Embryophyta (Phytozome Transcript Name): *Arabidopsis thaliana* (AT5G01220.1), *Populus trichocarpa* 1 (Potri.006G097600.1), *Populus trichocarpa* 2 (Potri.016G112600.1), *Oryza sativa* 1 (Os07g01030.1), *Oryza sativa* 2 (Os01g04920.1), *Oryza sativa* 3 (Os03g15840.1), *Selaginella moellendorffii* (170091), and *Physcomitrella patens* (Pp1s24_194V6.1).

**Figure 5:**
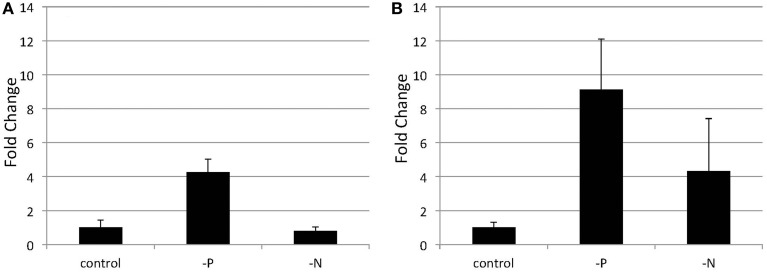
**Quantitative real-time PCR showing the induction of ***SQD2*** gene expression in *Nannochloropsis* after P or N starvation**. Logarithmic-phase cells were transferred to standard (control), -P and -N media and cultured for 4 days **(A)** or 6 days **(B)**. The values are normalized to the expression of TUB and the SQD2/TUB ratio under standard conditions was set as 1. Values represent the mean ± SD from three independent replicates.

Orthologs of the *SQD2* encoding the sulfoquinovosyl transferase (Yu et al., 2002) and a cyanobacterial counterpart, the *sqdX*, are highly conserved across different groups of algae and plants (Figure 4). We expected that the expression of homologs of the *SQD2* gene would be induced during P starvation. To determine the expression of SQD2 homologs in *Nannochloropsis* strain NIES-2145, we searched the *N. oceanica* genome (Vieler et al., 2012b) using a BLAST algorithm to identify amino acid sequences with similarity to *C. reinhardtii* and *Arabidopsis SQD2* genes. Using consensus sequences of the *SQD2* genes, we amplified the corresponding cDNA from *Nannochloropsis* strain NIES-2145. We sequenced the cDNA and found that *Nannochloropsis* strain NIES-2145 has a homolog of the *Arabidopsis SQD2* gene (Figure 4). Figure 4 is based on protein comparison. Expression of the *SQD2* gene homolog, as measured by qPCR, was induced after P deprivation in *Nannochloropsis* strain NIES-2145 (Figure 5). The *Chlamydomonas SQD2* promoter is useful for improving transgene expression in *C. reinhardtii* when P is depleted (Iwai et al., 2014). Therefore, we expected that the *Chlamydomonas SQD2* promoter would be available for inducing transgene expression to increase TAG production in *Nannochloropsis* under P-depleted conditions.

DGAT enzymes are important for TAG accumulation. During N starvation in *N. oceanica* IMET1, seven *DGAT* transcripts are up-regulated and six other *DGAT* transcripts are down-regulated (Li et al., 2014). However, it is unclear how *DGAT* transcripts are regulated during P starvation in *Nannochloropsis* strain NIES-2145. We therefore hypothesized that the overexpression of *C. reinhardtii DGTT4* in *Nannochloropsis* would be a convenient way to increase the lipid content or alter the lipid composition of these cells.

### Enhanced tag accumulation and changes in fatty acid profiles in *CrDGTT4*-overexpressing lines under P deprivation

**Figure 6:**
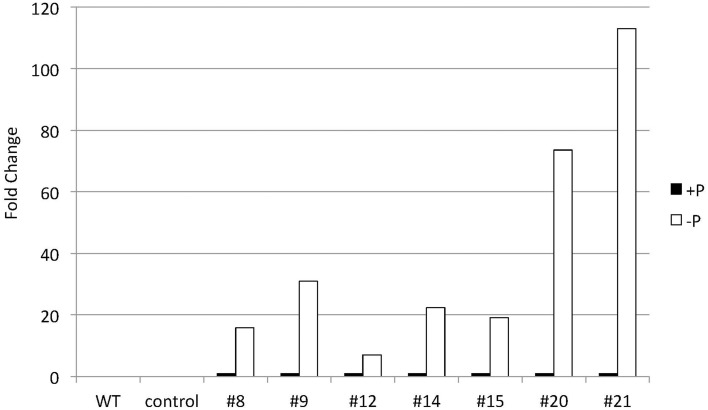
**Quantitative real-time PCR showing the induction of ***CrDGTT4*** gene expression in *CrDGTT4*-overexpressing cells after P deprivation**. Cells were cultured for 5 days. The relative levels of *CrDGTT4* mRNA overexpression in seven cell lines were assessed under standard (+P) and -P growth conditions. The values were normalized to the expression level of TUB. The CrDGTT4/TUB ratio under standard conditions was set as 1. The levels of the wild type (WT) and empty vector control (control) are below the detection threshold.

To explore CrDGTT4 as a tool to produce TAGs in *Nannochloropsis* cells, we expressed the CrDGTT4 coding sequence in *Nannochloropsis* under the control of the *CrSQD2* promoter, which increases transcription levels during P starvation in *C. reinhardtii* (Iwai et al., 2014). The relative level of overexpression of *CrDGTT4* was measured using qPCR. *CrDGTT4* mRNA levels in the overexpressing lines under P starvation were determined to be between 7- and 112-fold higher than in those under standard conditions (Figure 6), whereas no transcripts were detected in the wild-type and the empty vector controls. Thus, the *SQD2* promoter from *C. reinhardtii* is quite useful as a heterologous promoter in *Nannochloropsis* strain NIES-2145. Three overexpressing lines, #8, 9, and 21, were then selected for the subsequent experiments. To confirm the effect of *CrDGAT* gene overexpression, we compared the lipid content and fatty acid composition of the wild-type and overexpressing lines. All lines were cultured to the logarithmic phase prior to P depletion, as was done in the previous experiments.

**Figure 7:**
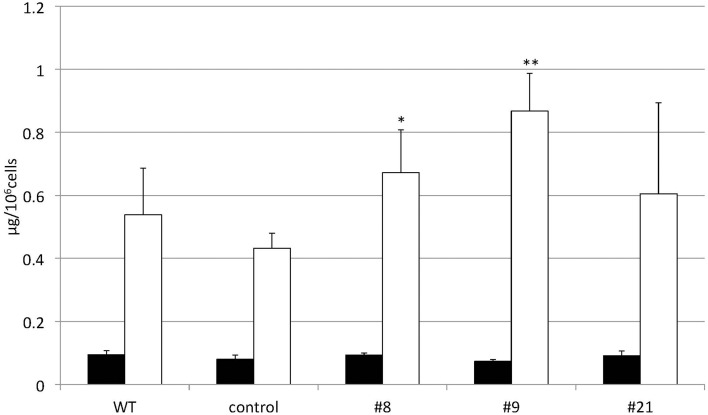
**Changes in the TAG content of ***CrDGTT4***-overexpressing cells in response to P starvation**. *Nannochloropsis* cells were transformed with pCrSQD2-CrDGTT4 to overexpress *CrDGTT4*. Cells cultured to a logarithmic phase under standard conditions were then inoculated into P-starved medium and cultured for 4 days. Three transformants, the empty vector control (control) and the wild type (WT) are shown. Values represent the mean ± SD from four independent replicates. Asterisks indicate a statistically significant difference compared with the empty vector control based on a two-tailed Student's *t*-test (^*^P < 0.05 and ^**^P < 0.01).

**Figure 8:**
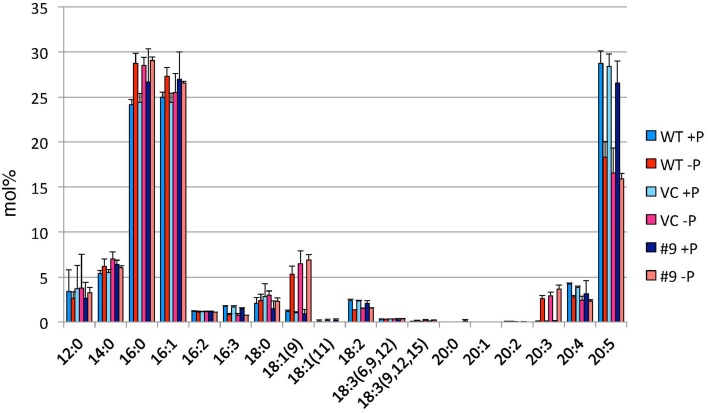
**Analysis of the fatty acid composition of total lipids in pCrSQD2-CrDGTT4 (#9), vector control (VC) and wild-type (WT) lines**. Cells were cultured in control (+P) or -P medium for 4 days. Values are the mean ± SD from three independent experiments.

**Figure 9:**
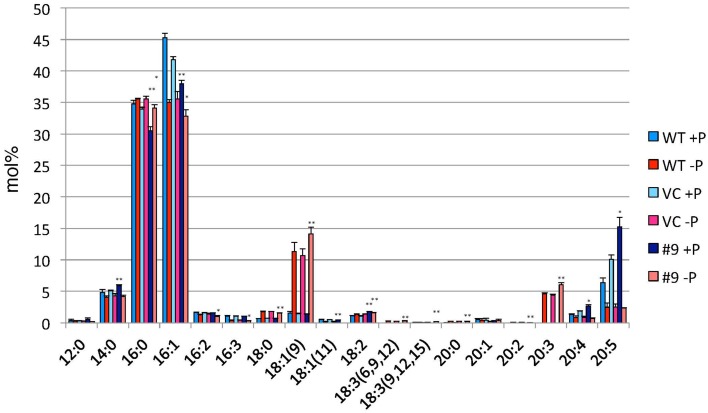
**Analysis of the fatty acid composition of the TAG fraction in pCrSQD2-CrDGTT4 (#9), the vector control (VC) and wild-type (WT) lines**. Cells were cultured in control (+P) or -P medium for 4 days. Values are the mean ± SD from three independent experiments. Asterisks indicate a statistically significant difference compared with VC based on a two-tailed Student's *t*-test (^*^P < 0.05 and ^**^P < 0.01).

TAG accumulation increased in *CrDGTT4*-overexpressing lines compared with the control and wild-type lines in P-depleted medium. *CrDGTT4*-overexpressing lines contained ~1.7-fold and ~1.3-fold more TAG per cell than the empty vector control cells at 4 and 6 days, respectively (Figure 7, Supplemental Figure 2). We next investigated if the fatty acid composition of total lipids and TAGs was altered in the *CrDGTT4*-overexpressing cell line. One of the overexpressing lines, line #9, was analyzed because of TAG accumulation most increased in this line. The major total fatty acids in either control or P-depleted medium were eicosapentaenoic (20:5 ω-3), palmitoleic (16:1 ω-7), and palmitic (16:0) acids (Figure 8). This agreed with previous analyses (Maruyama et al., 1986; Patil et al., 2007). The levels of 16:0, 16:1, oleic (18:1 ω-9), and dihomo-gamma-linolenic (20:3 ω-6) fatty acids increased, whereas 16:3, linoleic (18:2 ω-6), arachidonic (20:4 ω-6) and 20:5 fatty acids decreased under P starvation conditions (Figure 8). The degree of unsaturation for each fatty acid decreased as the unsaturated fatty acids were replaced with saturated fatty acids. As for the total fatty acid composition, no statistically significant differences could be observed among the wild-type, control and overexpressing lines (Figure 8). In the case of TAG fatty acids in the overexpressing line, the levels of 18:1 and 20:3 fatty acids were higher, whereas 16:0, 16:1, 16:2, and 16:3 fatty acids were lower than those in the wild-type or control lines during P starvation (Figure 9). The levels of 20:4 and 20:5 bound to TAGs (~0.8% and ~2.3%, respectively) were remarkably reduced under P starvation in comparison with the levels of these fatty acids in total fatty acids (~1–3% and ~15%, respectively; Figure 9). Under P starvation, the increase in the 20:3 fatty acid in the total lipids was a reflection of its increase in TAG. It is interesting to note that the level of 18:1 fatty acids was higher in the *CrDGTT4*-overexpressing cell line than in the control line under P starvation conditions (Figure 9). This is consistent with our previous report on *CrDGTT4*-overexpressing *C. reinhardtii* cell lines during P starvation (Iwai et al., 2014). These results showed that the *C. reinhardtii SQD2* promoter is useful as a heterologous promoter in the secondary endosymbiotic alga *Nannochloropsis* strain NIES-2145.

### P starvation did not cause significant changes in membrane lipid composition between the control line and overexpressing line

**Figure 10:**
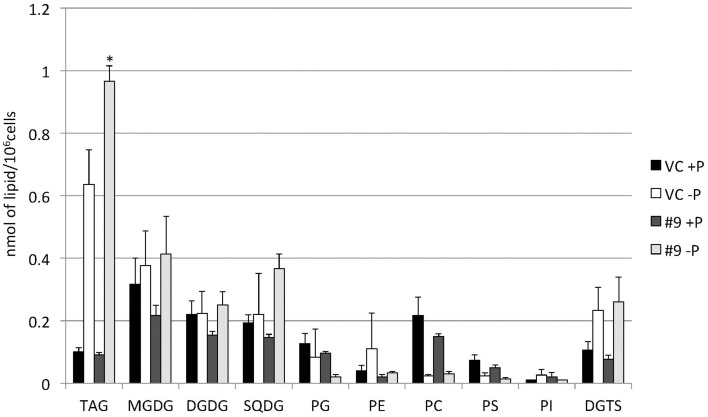
**Quantitative analysis of the various lipids**. Cells were cultured in control (+P) or -P medium for 4 days. Each lipid is expressed in nmol per 10^6^ cells. Values are the mean ± SD from three independent experiments. Asterisks indicate a statistically significant difference compared with VC based on a two-tailed Student's *t*-test (^*^P < 0.05).

**Figure 11:**
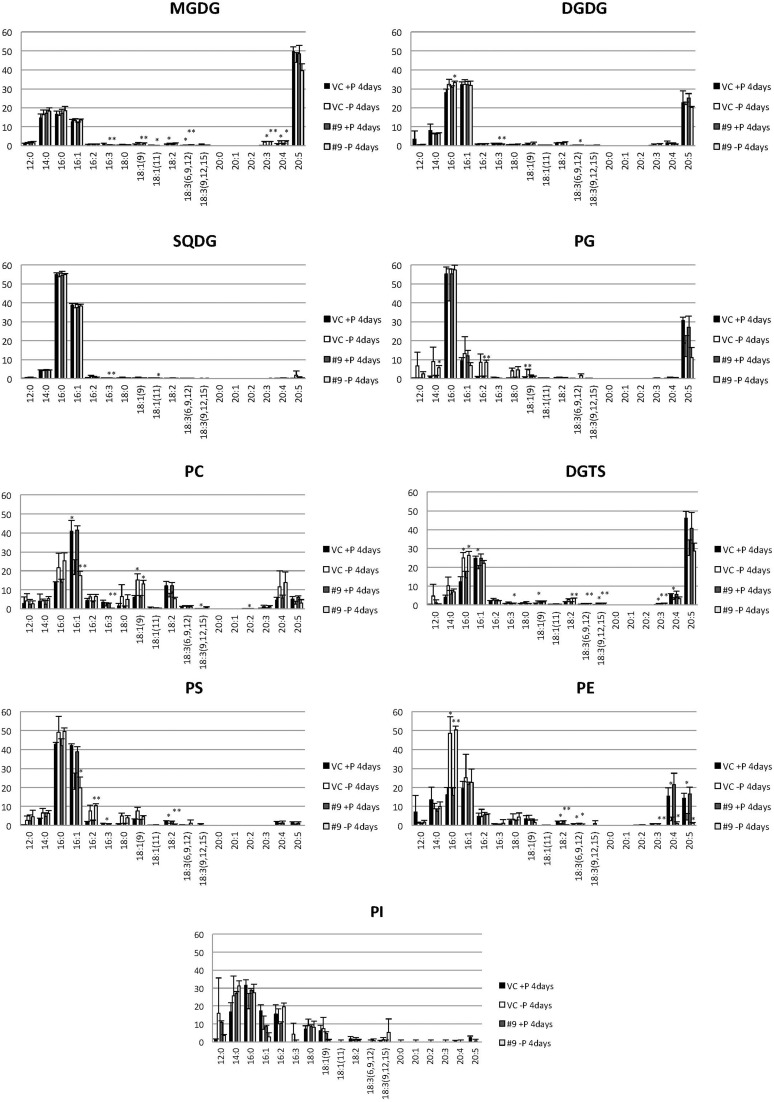
**Analysis of the fatty acid composition of the major lipids in pCrSQD2-CrDGTT4 (#9) and the vector control (VC) lines**. Cells were cultured in control (+P) or -P medium for 4 days. Black bars, the vector control (VC) 4 days in control medium; white bars, VC 4 days in -P; dark gray bars, pCrSQD2-CrDGTT4 (#9) 4 days in control medium; and light gray bars, pCrSQD2-CrDGTT4 (#9) 4 days in -P. Values are the mean ± SD from three independent experiments. Asterisks indicate a statistically significant difference compared with wild-type based on a two-tailed Student's *t*-test (^*^P < 0.05 and ^**^P < 0.01).

**Figure 12:**
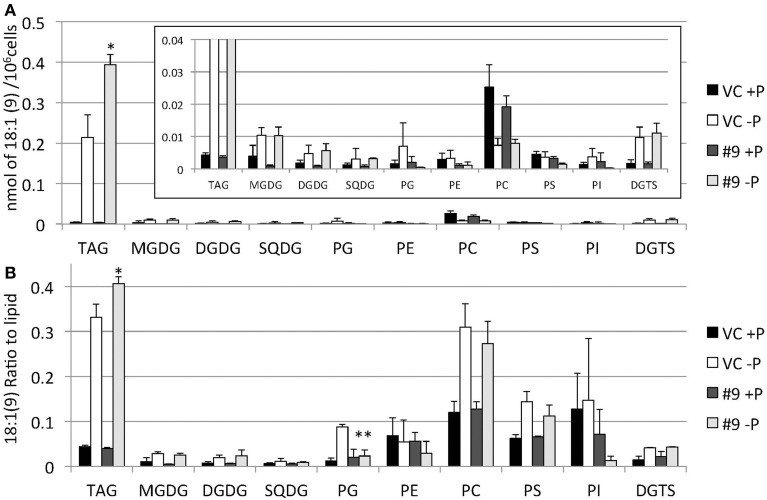
**Quantitative analysis and ratio of 18:1(9) in the various lipids**. Cells were cultured in control (+P) or -P medium for 4 days. **(A)** Each 18:1(9) in the various lipids is expressed in nmol per 10^6^ cells. **(B)** The y-axis is ratio of 18:1(9) in each lipid per 10^6^ cells. Values are the mean ± SD from three independent experiments. Asterisks indicate a statistically significant difference compared with VC based on a two-tailed Student's *t*-test (^*^P < 0.05 and ^**^P < 0.01).

**Figure 13:**
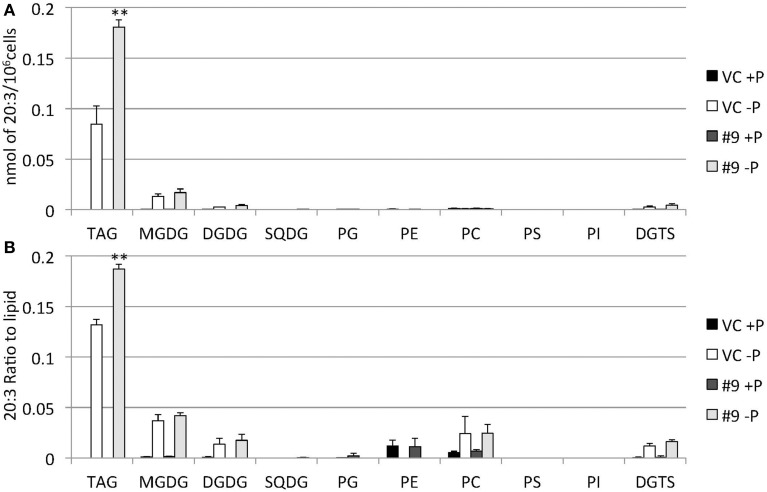
**Quantitative analysis and ratio of 20:3 in the various lipids**. Cells were cultured in control (+P) or -P medium for 4 days. **(A)** Each 20:3 in the various lipids is expressed in nmol per 10^6^ cells. **(B)** The y-axis is ratio of 20:3 in the various lipids per 10^6^ cells. Values are the mean ± SD from three independent experiments. Asterisks indicate a statistically significant difference compared with VC based on a two-tailed Student's *t*-test (^**^P < 0.01).

The overexpressing line showed alteration in TAG, whereas no serious alterations in the major membrane lipids compared with the control line under standard growth conditions or conditions of P stress, except for PS level (Figure 10, Supplemental Figure 3). PS decreased more in the overexpressing line than in the control line under P starvation (Supplemental Figure 3). We investigated whether the fatty acid composition of the major membrane lipids was altered in the *CrDGTT4*-overexpressing cell line (Figures 11–13, Supplemental Figures 4–7). During P-depleted conditions, the enhanced incorporation of 18:1(9) fatty acid was observed in the major plastidic membrane lipids MGDG, DGDG and PG and the extra-plastidic membrane lipids PC, PS, and DGTS (Figures 11, 12), whereas the enhanced incorporation of 20:3 fatty acid was observed in MGDG, DGDG, PC, and DGTS (Figures 11, 13). In the contrast, there were decreases of 18:1(9) and 20:3 fatty acids in PE. There was a global decrease in the long polyunsaturated fatty acid, 20:5 (Figure 11 and Supplemental Figure 4), and an increase in saturated fatty acids, for example 16:0 (Figure 11 and Supplemental Figure 5), during P-depleted conditions. Although large changes occur during P depravation, no significant differences could be observed in the major membrane lipids between the control line and overexpressing line, except for the decrease of 18:1 (9) fatty acid in the major plastidic membrane lipid PG.

## Discussion

Whereas TAG amounts increase under N starvation, MGDG, DGDG, SQDG, PG, and PI decrease in *C. reinhardtii* cells (Siaut et al., 2011; Sakurai et al., 2014) and the polar glycerolipid contents, represented mainly by MGDG, DGDG, and SQDG and phospholipids PE and PC, are reduced in *Nannochloropsis* strains (Simionato et al., 2013; Li et al., 2014; Martin et al., 2014). However, we found that SQDG and DGTS accumulated, replacing PG and PC, respectively, in *Nannochloropsis* cells under P starvation conditions (Figures 3, 10, Supplemental Figure 3). This result corroborates previous reports on P deprivation in several species (Benning et al., 1993, 1995; Essigmann et al., 1998; Sato et al., 2000; Khozin-Goldberg and Cohen, 2006). In *Monodus subterraneus*, which is a freshwater microalga in the class Eustigmatophyceae, DGTS and SQDG increased under P starvation conditions (Khozin-Goldberg and Cohen, 2006). In *A. thaliana*, SQDG is synthesized to replace PG in chloroplasts during P starvation so that the amount of anionic thylakoid lipid is maintained (Essigmann et al., 1998; Yu and Benning, 2003). In *C. reinhardtii*, mechanisms exist that maintain a constant total amount of SQDG and PG under sulfur- or P-limiting conditions (Sato et al., 2000). Therefore, our results suggest that SQDG substitutes for PG, to some extent, to sustain the functional activity of the decreased chloroplast membranes in *Nannochloropsis*, as in *C. reinhardtii*.

When the *SQD2* promoter is applied, total TAG levels per cell after 4 days of P starvation were ~1.7-fold greater in *CrDGTT4*-overexpressing lines compared with the control line (Figure 7). However, the difference between *CrDGTT4*-overexpressing lines and the control line after 6 days of P starvation was less than after 4 days (Supplemental Figure 2). During N starvation in *N. oceanica* IMET1, seven DGAT transcripts are up-regulated and six other DGAT transcripts are down-regulated (Li et al., 2014). Our results in *Nannochloropsis* might be explained by the up-regulation of *Nannochloropsis*'s own DGATs, particularly in later stages of P depletion. The levels of 18:1(9) and 20:3 fatty acids bound to TAGs increased during P starvation in comparison with the levels of these fatty acids under standard conditions (Figure 9). The levels of these two fatty acids in TAG molecular species were higher in the *CrDGTT4*-overexpressing cell line than in the control line during P starvation. This may reflect the specificity of CrDGTT4. In contrast to the TAG fraction, the 18:1(9) molecular ratio of PG decreased in the *CrDGTT4*-overexpressing cell line than in the control line during P starvation (Figures 11, 12). This fatty acid change may be associated with the preferential incorporation of the 18:1(9) molecular species into the TAG fraction.

It was unexpected that 20:3 fatty acid also increased in the *CrDGTT4*-overexpressing line (Figures 11, 13). This may have been due to the suppression of desaturation in very long chain fatty acids, like 20:4 and 20:5 (Figure 11 and Supplemental Figures 6, 7), because of accelerated TAG accumulation. Although the exact reason is unclear, 20:3 fatty acid tends to accumulate under P starvation conditions, but not under N starvation conditions (Simionato et al., 2013; Martin et al., 2014). This fact suggests that a specific pathway for fatty acid incorporation is activated during P starvation.

The SQD2 is highly conserved from red algae to plants, as are its primary structures (Figure 4), and the algal SQD2 is also conserved in secondary algae, including *Nannochloropsis*. This may be due to the importance of the acidic membrane lipid SQDG, which is common to various algal species. The importance of SQDG for photosystem II was demonstrated clearly for *C. reinhardtii* and the cyanobacterium *Synechocystis* sp. PCC6803 through the characterization of mutants from each species that are deficient in the ability to synthesize SQDG (Minoda et al., 2002; Sato et al., 2003; Aoki et al., 2004; Sato, 2004). Recent work on higher plant *SQD2* genes indicated that SQD2 is also involved with another acidic lipid, glucuronosyldiacylglycerol (Okazaki et al., 2013). It is possible that algal SQD2 also produces glucuronosyldiacylglycerol, which would thus be more important under P starvation conditions.

Our results showed that the *SQD2* promoter from the green alga *C. reinhardtii* is useful as heterologous promoter in *Nannochloropsis* strain NIES-2145 cells. The results of this study suggest that there is a common expression control system in a wide range of algal species, including primary and secondary microalgae, for adaptation to low P. This result, together with the occurrence of SQDG synthesis genes throughout algal species, suggests that a membrane remodeling system in chloroplasts under low-P stress is itself common among various microalgae and, thus, related genes and *cis*- and *trans*-acting elements are highly conserved. This also suggests that the *SQD2* promoter from *C. reinhardtii* could be applied in various algae.

*Arabidopsis SQD2* contains three PHR1-binding sequence (P1BS) motifs in its promoter. The PHR1 transcription factor binds to P1BS elements containing the consensus sequence “GNATATNC” in the promoters of a large number of P starvation–responsive genes (Franco-Zorrilla et al., 2004; Bustos et al., 2010; Pant et al., 2015). PHR1 is a well-described transcriptional regulator of P starvation in the MYB family. Most of the genes and their promoters that are involved in phospholipid degradation and glycolipid biosynthesis contain P1BS motifs in *Arabidopsis* (Pant et al., 2015). As there are no significant effects of a *PHR1* null allele on DGDG synthesis during P limitation in *Arabidopsis*, a role for PHR1 in lipid remodeling was thus excluded (Gaude et al., 2008). However, glycerolipids, including MGDG, DGDG and SQDG, their composition and the expression of most lipid-remodeling gene transcripts analyzed were all altered in the *phr1* mutant under P starvation when compared with the wild type (Pant et al., 2015). We used the *C. reinhardtii SQD2* gene (Cre01.g038550) promoter for *CrDGTT4* overexpression. Another *SQD2* gene homolog (Cre16.g689150) exists in *C. reinhardtii*. Both *C. reinhardtii SQD2* genes were up-regulated under P starvation (data not shown). We searched the promoter regions of these genes, and they do not contain P1BS motifs in their promoters but have binding motifs for many other transcription factors. *C. reinhardtii SQD2* has 14 MYB or MYC binding domains in its promoter region, whereas *Nannochloropsis oceanica SQD2* has 12 MYB or MYC binding domains in its promoter region (Supplemental Figure 8). There is no reliable evidence revealing which motif offers the most effective transcriptional regulation under P starvation. Further work is necessary to understand the regulation of lipid remodeling during P stress in algae.

## Author contributions

MI prepared samples of *Nannochloropsis* strain NIES-2145 for each experiment. KH performed *in silico* analysis. YS performed GC-MS analysis. MI, KH, YS and HO wrote the manuscript. MS and HO planned the project.

## Conflict of interest statement

The authors declare that the research was conducted in the absence of any commercial or financial relationships that could be construed as a potential conflict of interest.

## Supplementary material

Supplemental Figure 1 **Changes in the TAG content of *Nannochloropsis* in response to N or P starvation**. Cells cultured to the logarithmic phase under f/2 medium were then inoculated into f/2, -N or -P medium and cultured for 13 d.

Supplemental Figure 2 **Changes in the TAG content of *CrDGTT4*-overexpressing cells in response to P starvation**. *Nannochloropsis* cells were transformed with pCrSQD2-CrDGTT4 to overexpress *CrDGTT4*. Cells cultured to the logarithmic phase under standard conditions were then inoculated into control (+P) or -P medium and cultured for 6 days (6 d). The transformants (#8, #9, and #21), the empty vector control (VC) and the wild type (WT) are shown. Values represent the mean ± SD from three independent replicates.

Supplemental Figure 3 **Changes in major lipid classes in P-starved cells**. Black bars, the vector control (VC) 4 days in control medium (+P); white bars, VC 4 days in -P; dark gray bars, pCrSQD2-CrDGTT4 (#9) 4 days in control medium (+P); and light gray bars, pCrSQD2-CrDGTT4 (#9) 4 days in -P. Values are the mean ± SD from three independent experiments. Asterisks indicate a statistically significant difference compared with VC based on a two-tailed Student's *t*-test (^*^P < 0.05). Small letters indicate statistically significant difference compared with the control medium based on a two-tailed Student's *t*-test (^a^*P* < 0.05 and ^b^*P* < 0.01).

Supplemental Figure 4 **Quantitative analysis and ratio of 20:5 in the various lipids**. Cells were cultured in control (+P) or -P medium for 4 days. **(A)** Each 20:5 in the various lipids is expressed in nmol per 10^6^ cells. **(B)** The y-axis is ratio of 20:5 in each lipid per 10^6^ cells. Values are the mean ± SD from three independent experiments. Asterisks indicate a statistically significant difference compared with VC based on a two-tailed Student's *t*-test (^*^*P* < 0.05).

Supplemental Figure 5 **Quantitative analysis and ratio of 16:0 in the various lipids**. Cells were cultured in control (+P) or -P medium for 4 days. **(A)** Each 16:0 in the various lipids is expressed in nmol per 10^6^ cells. **(B)** The y-axis is ratio of 16:0 in each lipid per 10^6^ cells. Values are the mean ± SD from three independent experiments. Asterisks indicate a statistically significant difference compared with VC based on a two-tailed Student's *t*-test (^*^*P* < 0.05).

Supplemental Figure 6 **Quantitative analysis and ratio of 16:1 in the various lipids**. Cells were cultured in control (+P) or –P medium for 4 days. **(A)** Each 16:1 in the various lipids is expressed in nmol per 10^6^ cells. **(B)** The y-axis is ratio of 16:1 in each lipid per 10^6^ cells. Values are the mean ± SD from three independent experiments. Asterisks indicate a statistically significant difference compared with VC based on a two-tailed Student's *t*-test (^*^*P* < 0.05).

Supplemental Figure 7 **Quantitative analysis and ratio of 20:4 in the various lipids**. Cells were cultured in control (+P) or -P medium for 4 days. **(A)** Each 20:4 in the various lipids is expressed in nmol per 10^6^ cells. **(B)** The y-axis is ratio of 20:4 in each lipid per 10^6^ cells. Values are the mean ± SD from three independent experiments. Asterisks indicate a statistically significant difference compared with VC based on a two-tailed Student's *t*-test (^*^*P* < 0.05).

Supplemental Figure 8 **MYB/MYC binding motif in the *SQD2* promoter region of *Nannochloropsis oceanica* and *Chlamydomonas reinhardtii***. Yellow character, CTGTTA (+) strand MYB/MYC binding motif; red character, (+) and (−) strand MYB/MYC binding motif; blue character, (−) strand MYB/MYC binding motif; green character, CDS; pale green character, UTR region.

Supplemental Table 1 **Primer sequences**.
