# Supplementary figures and images for: Manipulation of oil synthesis in Nannochloropsis strain NIES-2145 with a phosphorus starvation–inducible promoter from Chlamydomonas reinhardtii
Source: Front Microbiol. 2015 Sep 7;6:912. doi: 10.3389/fmicb.2015.00912 (PMC4561341; doi:10.3389/fmicb.2015.00912)

# Sup fig1

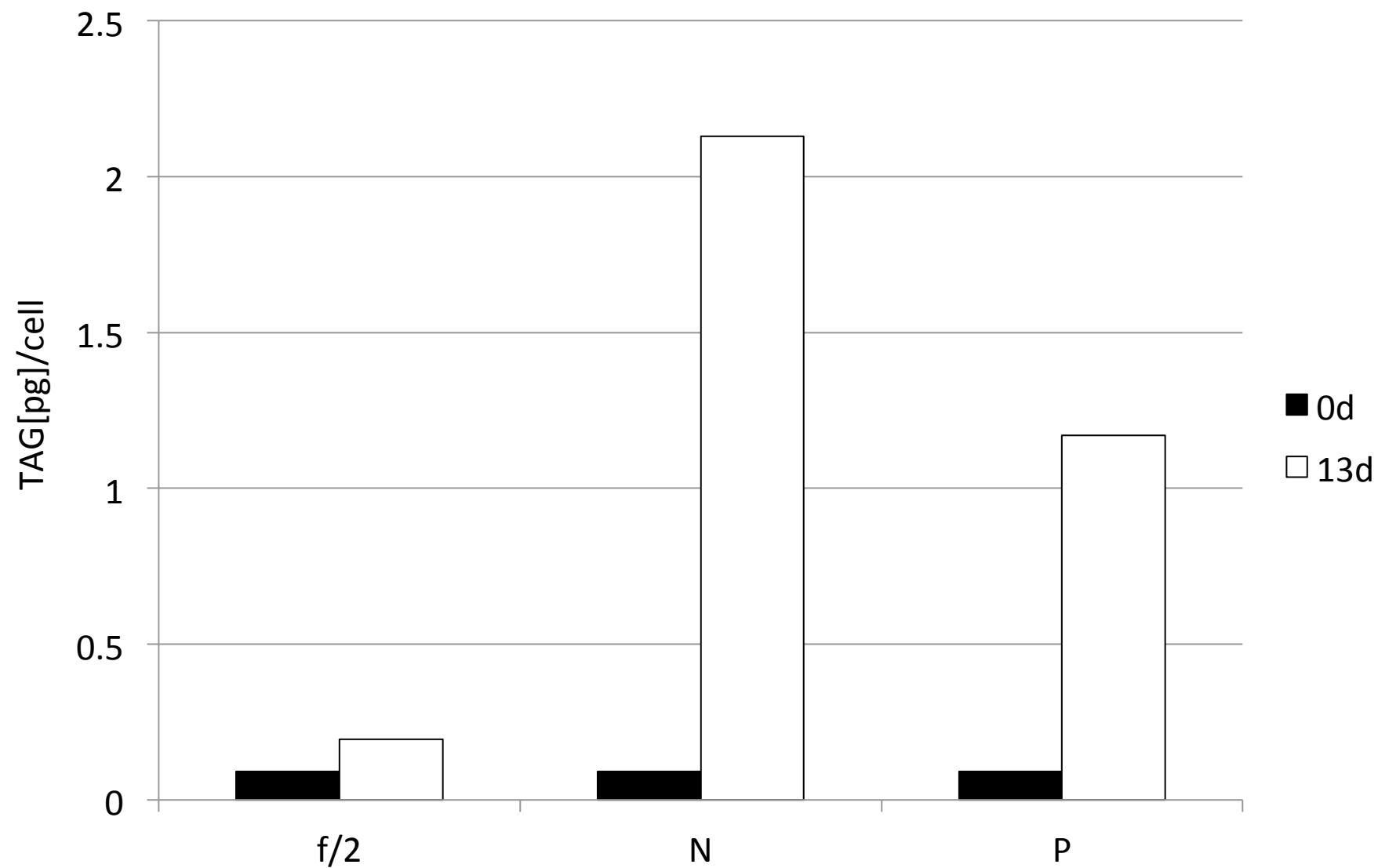

Supplement: Supplemental Figure 1 — Changes in the TAG content of Nannochloropsis in response to N or P starvation. Cells cultured to the logarithmic phase under f/2 medium were then inoculated into f/2, -N or -P medium and cultured for 13 d. [file Image1.PDF]

# supplemental fig 2

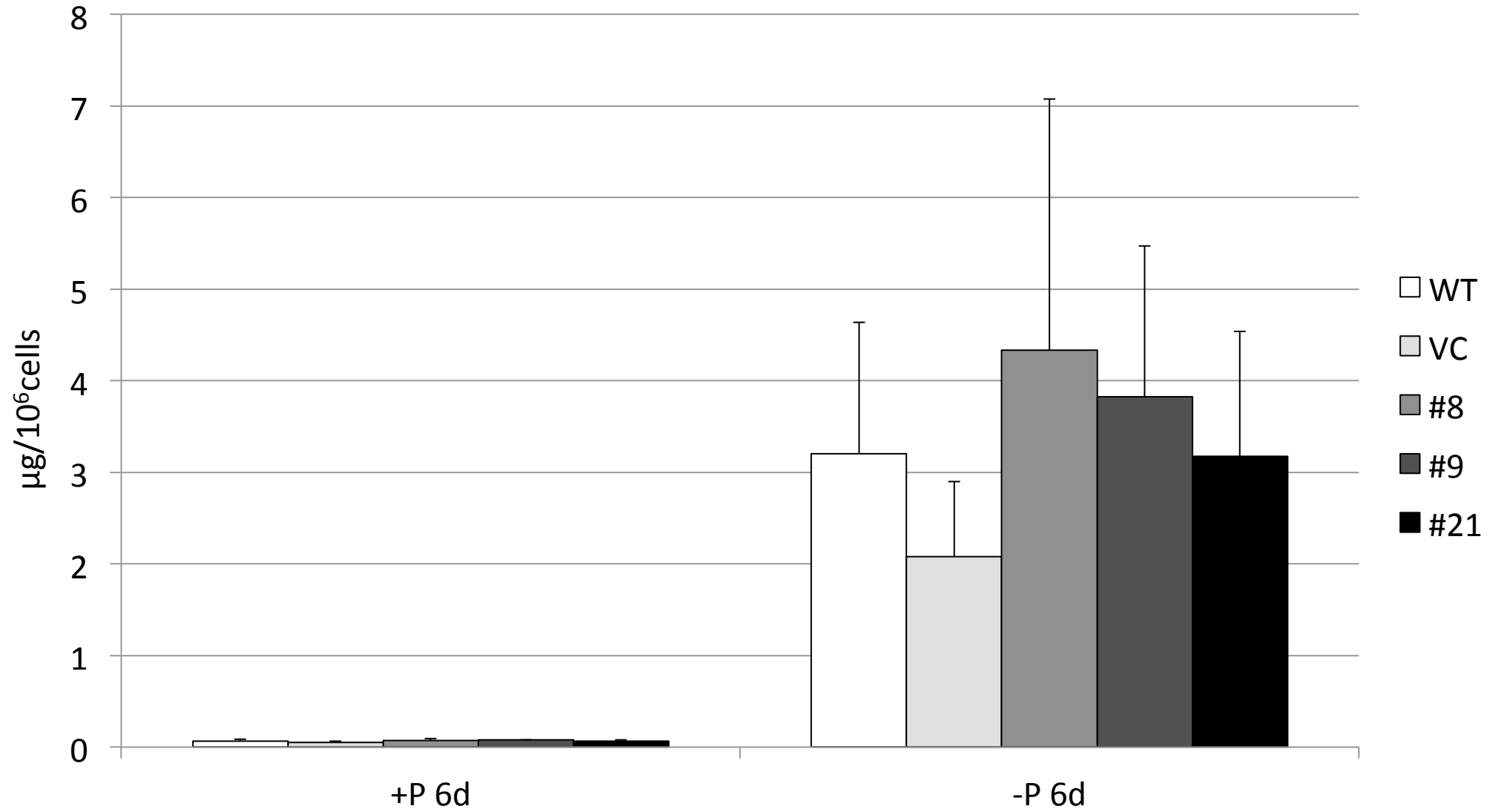

Supplement: Supplemental Figure 2 — Changes in the TAG content of CrDGTT4-overexpressing cells in response to P starvation. Nannochloropsis cells were transformed with pCrSQD2-CrDGTT4 to overexpress CrDGTT4. Cells cultured to the logarithmic phase under standard conditions were then inoculated into control (+P) or -P medium and cultured for 6 days (6 d). The transformants (#8, #9, and #21), the empty vector control (VC) and the wild type (WT) are shown. Values represent the mean ± SD from three independent replicates. [file Image2.PDF]

# Sup Figure 3

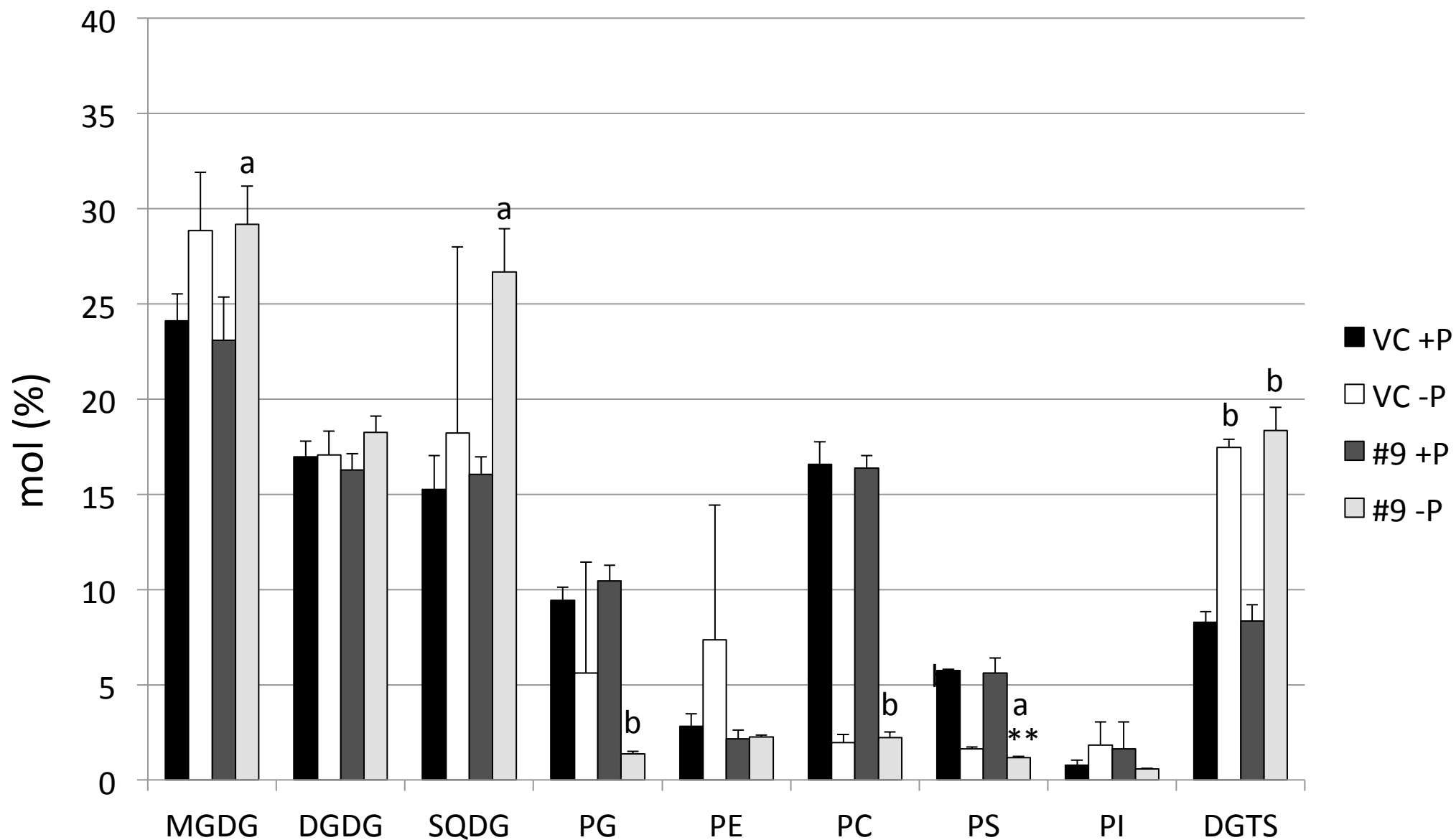

Supplement: Supplemental Figure 3 — Changes in major lipid classes in P-starved cells. Black bars, the vector control (VC) 4 days in control medium (+P); white bars, VC 4 days in -P; dark gray bars, pCrSQD2-CrDGTT4 (#9) 4 days in control medium (+P); and light gray bars, pCrSQD2-CrDGTT4 (#9) 4 days in -P. Values are the mean ± SD from three independent experiments. Asterisks indicate a statistically significant difference compared with VC based on a two-tailed Student's t-test (*P < 0.05). Small letters indicate statistically significant difference compared with the control medium based on a two-tailed Student's t-test (aP < 0.05 and bP < 0.01). [file Image3.PDF]

# Sup Figure 4

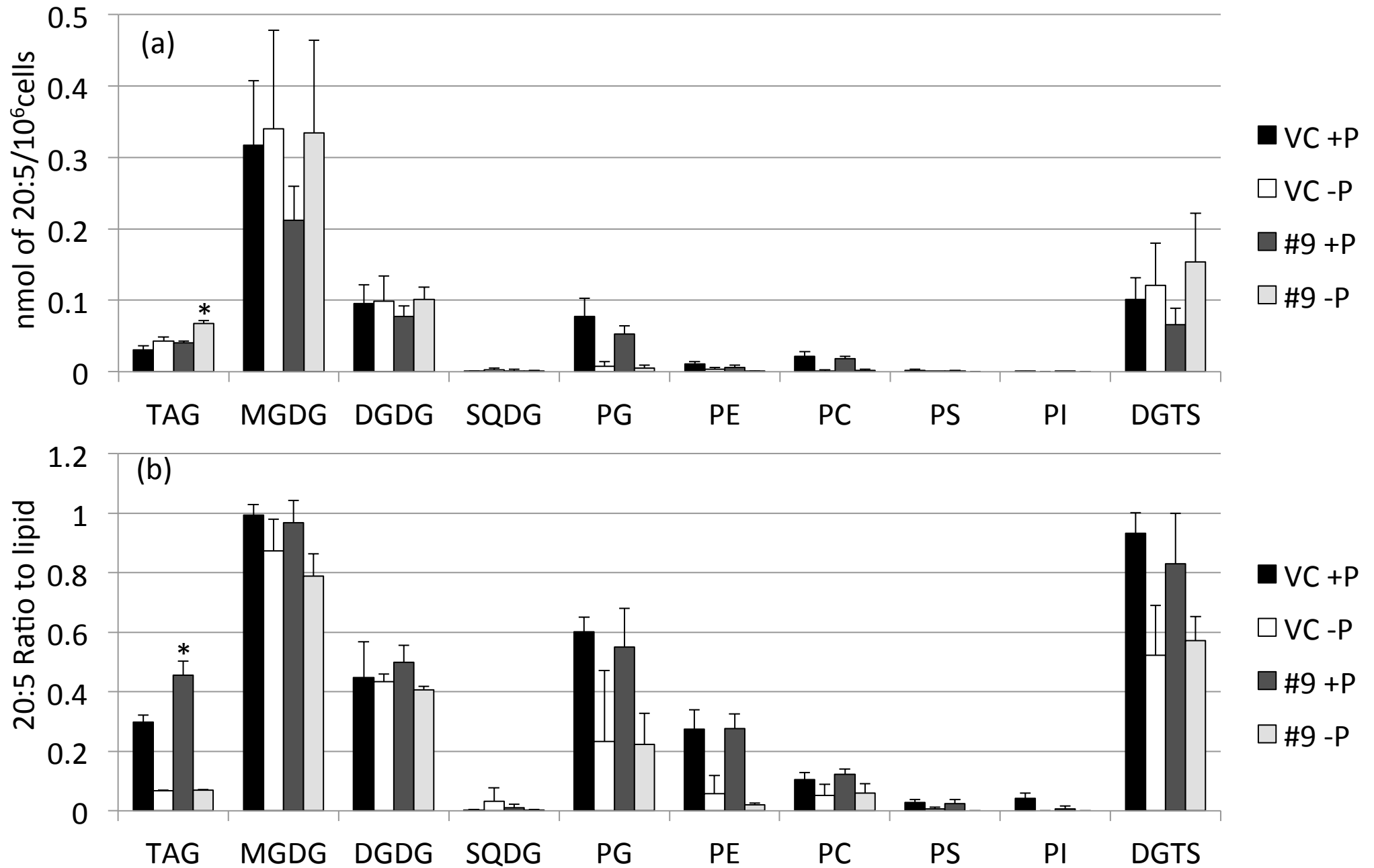

Supplement: Supplemental Figure 4 — Quantitative analysis and ratio of 20:5 in the various lipids. Cells were cultured in control (+P) or -P medium for 4 days. (A) Each 20:5 in the various lipids is expressed in nmol per 106 cells. (B) The y-axis is ratio of 20:5 in each lipid per 106 cells. Values are the mean ± SD from three independent experiments. Asterisks indicate a statistically significant difference compared with VC based on a two-tailed Student's t-test (*P < 0.05). [file Image4.PDF]

# Sup Figure 5

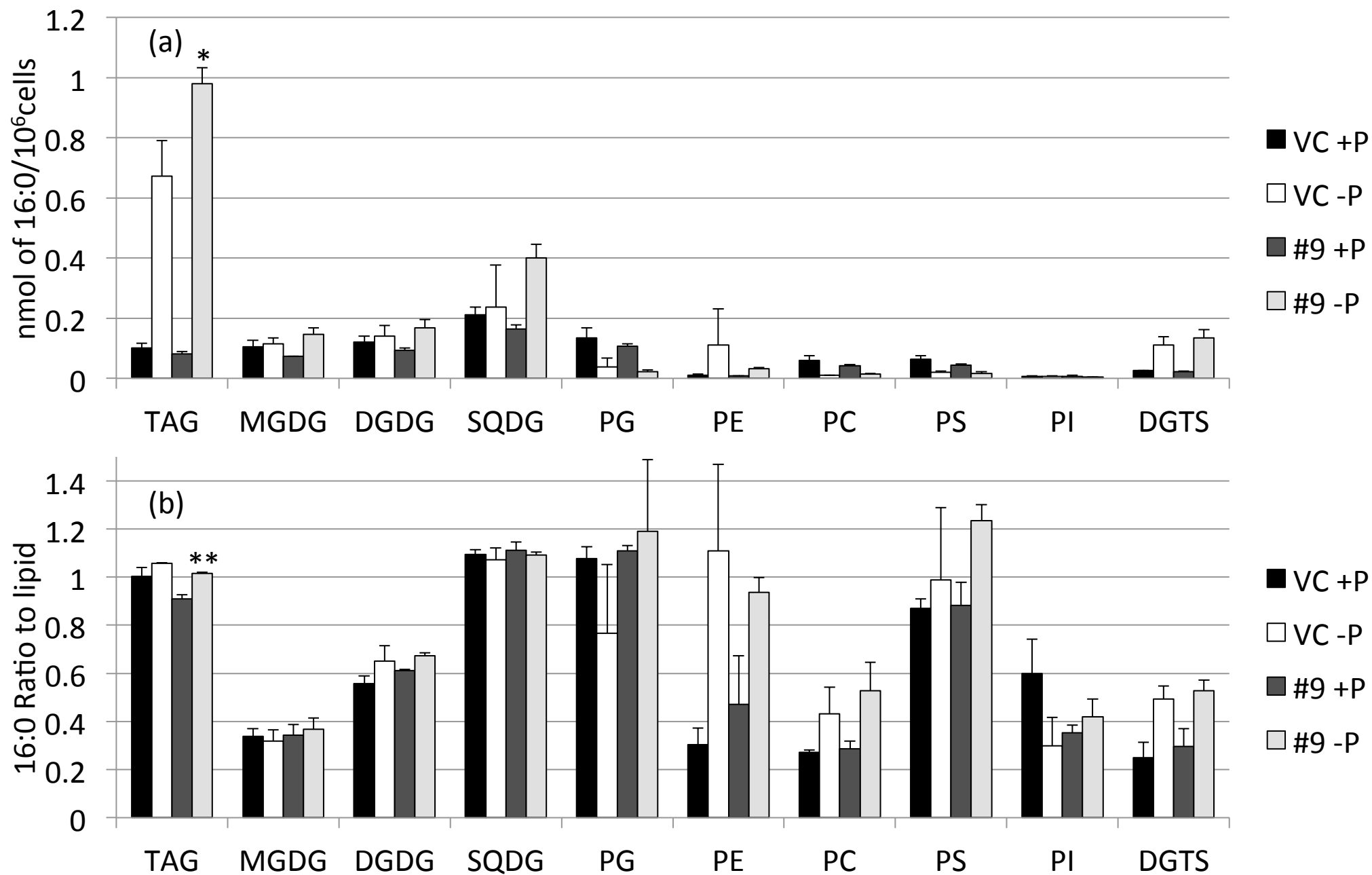

Supplement: Supplemental Figure 5 — Quantitative analysis and ratio of 16:0 in the various lipids. Cells were cultured in control (+P) or -P medium for 4 days. (A) Each 16:0 in the various lipids is expressed in nmol per 106 cells. (B) The y-axis is ratio of 16:0 in each lipid per 106 cells. Values are the mean ± SD from three independent experiments. Asterisks indicate a statistically significant difference compared with VC based on a two-tailed Student's t-test (*P < 0.05). [file Image5.PDF]

# Sup Figure 6

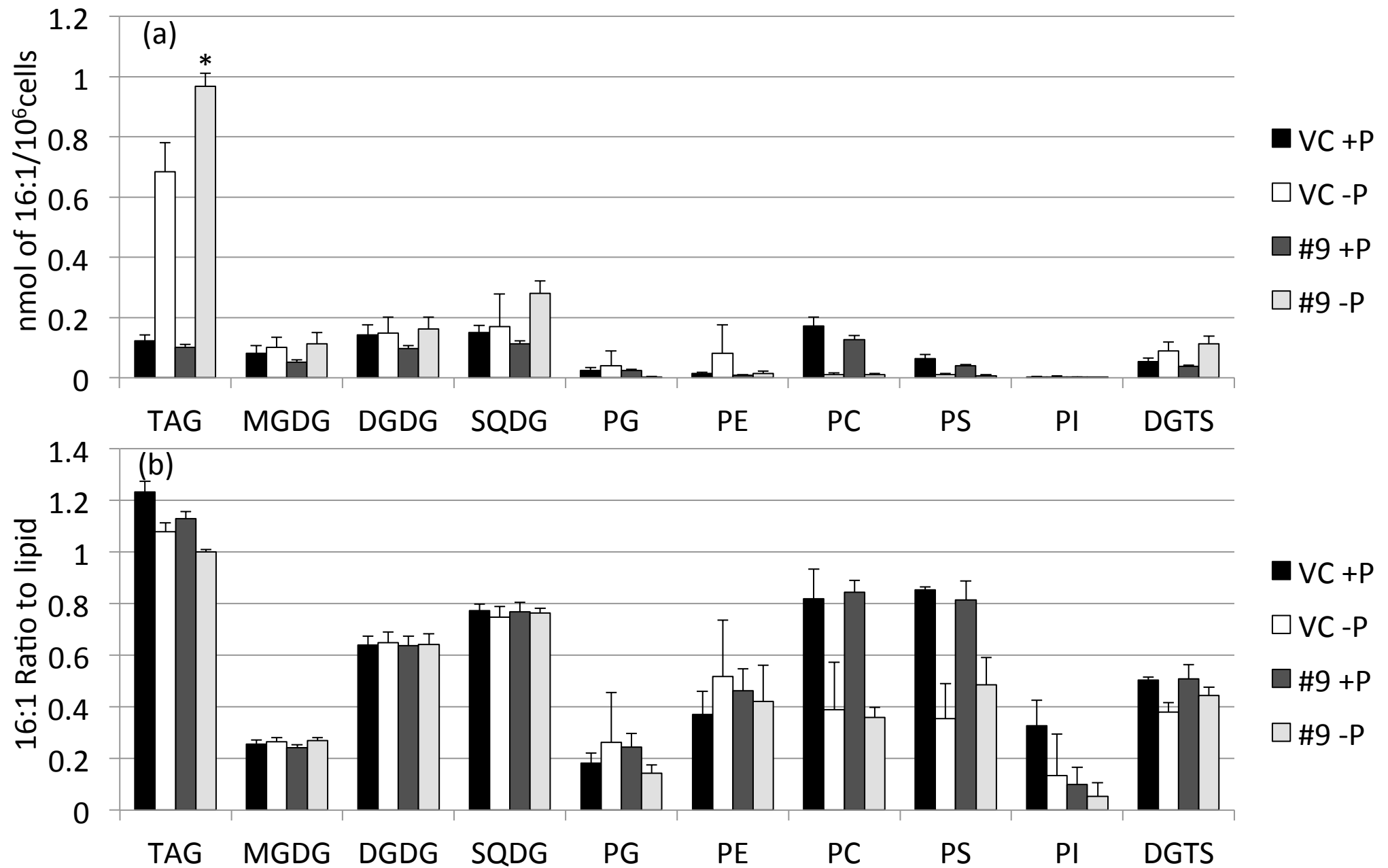

Supplement: Supplemental Figure 6 — Quantitative analysis and ratio of 16:1 in the various lipids. Cells were cultured in control (+P) or –P medium for 4 days. (A) Each 16:1 in the various lipids is expressed in nmol per 106 cells. (B) The y-axis is ratio of 16:1 in each lipid per 106 cells. Values are the mean ± SD from three independent experiments. Asterisks indicate a statistically significant difference compared with VC based on a two-tailed Student's t-test (*P < 0.05). [file Image6.PDF]

# Sup Figure 7

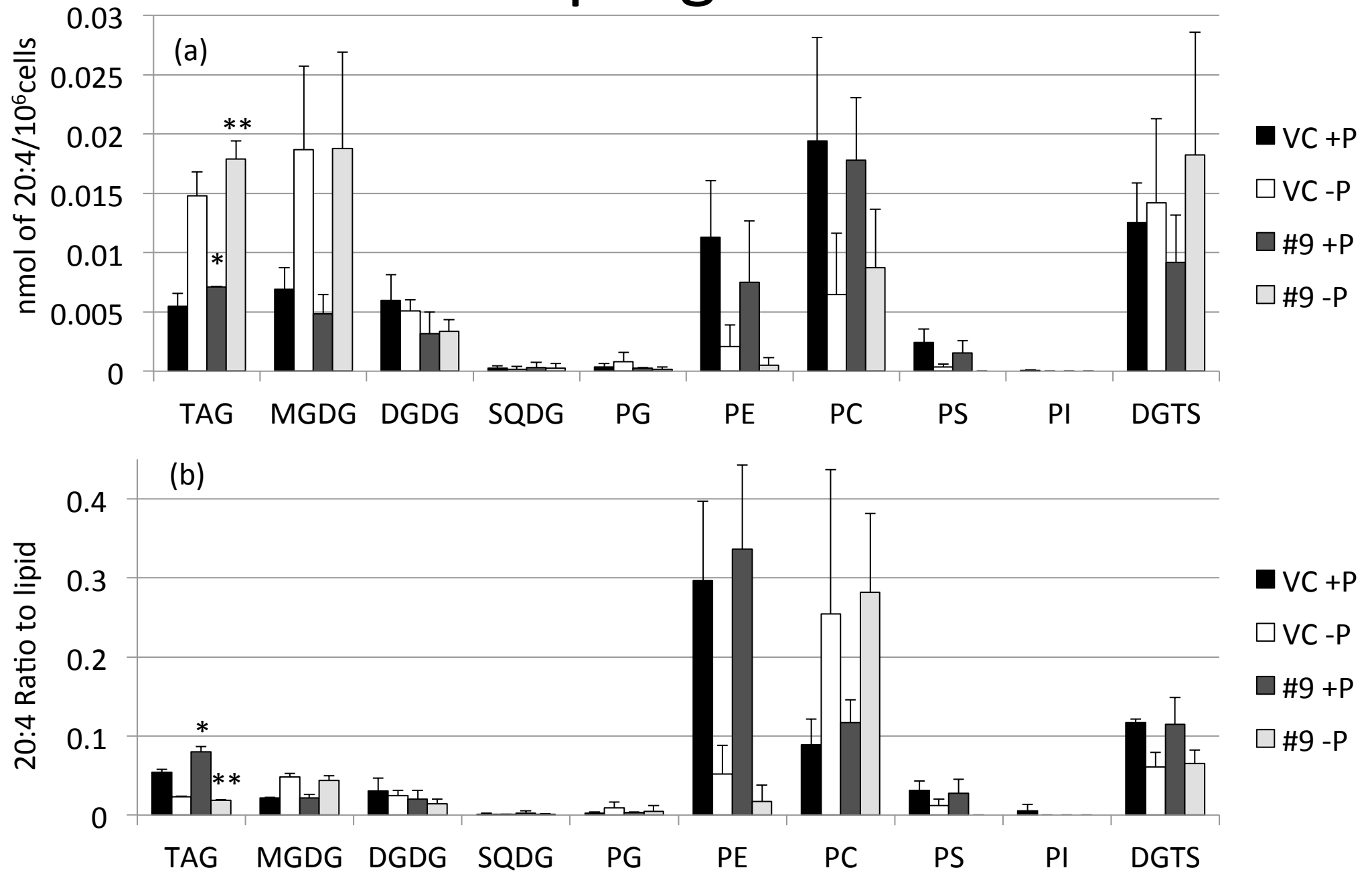

Supplement: Supplemental Figure 7 — Quantitative analysis and ratio of 20:4 in the various lipids. Cells were cultured in control (+P) or -P medium for 4 days. (A) Each 20:4 in the various lipids is expressed in nmol per 106 cells. (B) The y-axis is ratio of 20:4 in each lipid per 106 cells. Values are the mean ± SD from three independent experiments. Asterisks indicate a statistically significant difference compared with VC based on a two-tailed Student's t-test (*P < 0.05). [file Image7.PDF]
