# Supplementary material for: Manipulation of oil synthesis in Nannochloropsis strain NIES-2145 with a phosphorus starvation–inducible promoter from Chlamydomonas reinhardtii
Source: Front Microbiol. 2015 Sep 7;6:912. doi: 10.3389/fmicb.2015.00912 (PMC4561341; doi:10.3389/fmicb.2015.00912)
Supplement: Supplemental Figure 8 — MYB/MYC binding motif in the SQD2 promoter region of Nannochloropsis oceanica and Chlamydomonas reinhardtii. Yellow character, CTGTTA (+) strand MYB/MYC binding motif; red character, (+) and (−) strand MYB/MYC binding motif; blue character, (−) strand MYB/MYC binding motif; green character, CDS; pale green character, UTR region. [file Image8.PDF]

Chlamydomonas reinhardtii SQD2 upstream

Sup Figure 8

GTGCCCTCCTTCACATACACCTCGCCCGCCGCCTCCACCAGCACCGCCCGCGCCTCCGCCCGCCCCGTCACCAGCGCCACCCGCTCCCCGCCCAGCAGCAGCCCCACCACCGGGCCGTAGGTGGCCGTGGCGTCGGTCAGG

AAGCGGAGCGGGTCGGTGAGCAGAGGCAGCGTCTGGTCTCCGGAGGGGCCGGCGGGGAAGGCGGGCGGCGGCGGGGAGGACAGGCGCTGGAGCCGCGTGGCGGCGCCGGCGGCGGCGGCGGTGAGCTGGGCGGCGGCGGCG

GAGATCTGCGAGGCTGAGGCCTGCAGCAGGCTGAGCCACAGGTCCAGGCGGAGGTCGGAGGTGGCGGGCGGCGACGCCGCCAAAGGTGCGAAGGGGGCAGCGGAGGTGGAGGAGGAGGAGGTGGTGGTGGCGGGATAG

TTGTAGCTGTAGCTTTTGCCGGGCGCGGGCGAGGGCGGCAGCCCGTTGGGGGCGGTGTTGCCGTTTGCCGAGGCCACGGGGCGCGGGGGCGCGGTGGTGTGGCCTTGGCTGGGTGTGGCGGCGGCGAGGACGCCCCTGCGC

CGGTCGCTGCGGTGTCGGCAGCGGCTGCTGGACGCCGCTCCTGGTGCCGCGCAGGTCTGCACGGCCGTGTACAGGCTATTGGGGCCCGGCCCTGGCGCCAGTTCCGGGGCTGAGCAAAGTAAGCCCAAGGTGGAGGCCGCT

GATGGCGCAGATGCCTGAGGGTAAACTGCGCCCTGGCCTGCATGGCGGCTGGCTCGGTTGGCGGTTGCGGGCACGGTCGCGGTGACGGTGGCTCCGACAGGCTCAAATGGCCGAACGCCGCGCGGCAGGCGCGTCGCCGGT

AATGCCACTACCATATTTTCACTTTTAATTCCTCCAACTGCAGTGGCAACGTTGATTTGGGTTCTCAACAGGCAACAATCGAGGACTTGCAGACCGAAACGCTGGGTTTGTACCCCTGATGCGGTAATAATATTTTAAGTT
<-Cre01.g038500.t1.2 CDS

GAGCATATAGCAAGACCTGCAATCAAAGGGAGTCAACAACCGCTTTGTTGGGCGGGCCTCATGGCCTGCAACGTGCATCGGACCATCGCGTCTCTTTCGCCGCTTCCACTAGGGTTTGCGCCAGAGAACAAGTGAGCCATA
<-Cre01.g038500.t1.2 CDS

TGTGATTTTGTGCGTGACCAAAGGTGAGCTCACTTGCAGCATGCTCTGCAAGCCTCCCGCCAAGGCAAGCATAAACCGCTTCGGACGGCTCCTCGTTCTCCTGGACTTATTGCAGGCTGGTCTACGTGCGCAATTGAGCTG
SQD2 transcript->

CGTACATTGAAGGCCACGCGCGGCGCCGGCGAAGCCCGACGCAAGCTGTGATACCGGAATATCAGTATCAGCGCGTCGATGCGCTACTTTTAGAGGGCCAAGAGCGACAATGAACACACACCTCAACTCTTCGCTGTCCAA
Cre01.g038550 SQD2 CDS ->

TGGCGCGTCGACCGGCGGGTCCTCGAGG...

Nannochloropsis oceanica CCMP1779 SQD2 upstream

ATTATCTCTCCTCCTTCACATCACACGTATTTCGTATAGAGGCAACACACAACCAAGTCAGCAATGTTCATCAAGAAGCTGCCTGCACTCCTTGTGCTTGCTTTATGCGCCACCAACGCCGCTGCCGCCGGTTACATCAAG
NannoCCMP1779_4366-mRNA-1 ->

ACGTTTAACGCCTCCAACTACGCCCTGACAACATATGATGCCGCCGCTGAAAAGCCCCTCCGGGTGAGGCCCAACACCGTGTACACGATCGACAAGTTCCTCGACGAAGACTTCCACCTCGTTATTCAGTGTCCTCAGCCC

AAGAATAACCGCATGAGGAAGAAGGGTTTCATCACCTATTTGAGCTATTACCAAGGCATTGACGAAAGTGCCGTCGAGGTCTATGAGGACGGCAGCGACATCGCACAAGCTGAGATGTATAGCATCATTGATGCTGGGTAC

GGGTCTTTGCTTGTACTGTTCTGCAACAATGGCAAGCTTACCTTGAACTACAACTATGCCGGCTAATGAGAGAACGGGATGCTGAATAATATAATTGAATCAACGGACCAGCTTCTCATTCCAATCTCCATGTTTTTGCTG

TTATGTGTCGTGTTGTGTTGCGGGGATGTGTGTGTCTCTCGCGGCGTCCTGTTAATTCTATTTCTGCGCGGGAATGTGAGCATATAATTCAGGGACCGGTGAGAGCCCGGCAACCCCATCAGAAGCCTTGATGGATGAACA

ATCAGATTGTAACGAGTGGCGAAGTTGTGAAAGAATTAAAAAGAAGGTGCCGAAGATGAAAATTTAGCTAATGCTTTCTTCCCCCCCCCCCCAGTAGAAGATCACCGAGCCATCTGTAAGAAGGCCATAAGCCACCAAGCT

GATGTTTGCTGGGCGTGTAGATATGGATTATCTCACAGAGTGACAGGAGTCTGATATCAAGATAGAGGAGTCTCAAGACTTCTGGAGATCGCACACCGTGTCTCCTCGCAAGGGATGCAGTGTTGGCCAGCTTCTGTAGAA

AAGGTGGGCATAAGTTAAGCACAATTTGCTGTCATCGTCGTAATTGCTGCTTGTTTTCCTCCGCACAACTAAATCCCACTGTTACATAGTAAATTTCATGTCCTTTTCCCCGTTTTTGCACGGTGTTACATTCGCGCCAGA

TTGCCTTCATGAACGAATCCTTGTGTTCCGATTCTAAAATCACCACCCACCCACCCATCCCACCACCAGAGGCCTCAGAGCCCCAACCACACGTGCTAGGTTACTCAATCATGGTGTCGTCCACAGGTTGTGGATGTCGCA

TGGGTGAAAATGACCAGGCCGCGACCTCCACCGCTTCCACTTGGACGCCGATCATGTTGATAG...

NannoCCMP1779_4348 SQD2 CDS ->
