# Supplementary material for: Manipulation of oil synthesis in Nannochloropsis strain NIES-2145 with a phosphorus starvation–inducible promoter from Chlamydomonas reinhardtii
Source: Front Microbiol. 2015 Sep 7;6:912. doi: 10.3389/fmicb.2015.00912 (PMC4561341; doi:10.3389/fmicb.2015.00912)
Supplement: Supplemental Table 1 — Primer sequences. [file Table1.PDF]

SUPPLEMENTAL TABLE 1 | Primer sequences

Gene	primer name	primer sequence (5′-3′)
Tub	NannoTUB_realRT_F	AGCATGGCATTGACTCCACC
	NannoTUB_realRT_R	AACGGCCTCGTTGTAGTACACG
NoSQD2	NoSQD2_realRT_F	TCCCTTGCTTACTGCTCTGG
	NoSQD2_realRT_R	GATTCGCGTAGCCGCTTA
NoSQD2	NoSQD2_CDS_F	CATGGGTGAAAATGACCAGGCCG
	NoSQD2_CDS_R	TTCAAACAACTCACGCCCTCTCC
CrDGTT4	DGTT4_realRT_F	AAAGCTGCGAAACGTGGTG
	DGTT4_realRT_R	GGCCCCAAACAGGTAGAACA
